# Penile vascular status in young men living with HIV experiencing erectile dysfunction: A comparative cross‐sectional pilot study

**DOI:** 10.1111/andr.70050

**Published:** 2025-04-23

**Authors:** Giorgio Tiecco, Andrea Delbarba, Cosimo Colangelo, Marco Di Gregorio, Paolo Facondo, Matteo Riva, Carlo Cappelli, Emanuele Focà, Francesco Castelli, Eugenia Quiros‐Roldan

**Affiliations:** ^1^ Department of Clinical and Experimental Sciences SD of Infectious and Tropical Diseases University of Brescia and ASST Spedali Civili di Brescia Brescia Italy; ^2^ Department of Clinical and Experimental Sciences SSD of Endocrinology and Metabolism University of Brescia and ASST Spedali Civili di Brescia Brescia Italy; ^3^ Department of Medical and Surgical Specialties Radiological Sciences and Public Health University of Brescia, ASST Spedali Civili of Brescia Brescia Italy

**Keywords:** andrology, erectile disfunction, HIV, IIEF‐5, intima‐media thickness

## Abstract

**Background:**

Erectile dysfunction in people living with HIV is a multifactorial disease, but the role of penile vascular status assessed by dynamic penile color Doppler echography is underexplored.

**Objectives:**

This study assessed penile vascular status in young males living with HIV experiencing erectile dysfunction, comparing them to HIV‐negative controls stratified into young (<50 years) and middle‐aged (51–60 years).

**Materials and methods:**

This monocentric, comparative cross‐sectional study included young males living with HIV (18–50 years) on antiretroviral therapy for >12 months and HIV‐negative individuals presenting with erectile dysfunction. We used dynamic penile color Doppler echography to evaluate penile vascular parameters such as peak systolic velocity, intima‐media thickness, and end‐diastolic velocity. Statistical analyses, including *k*‐means clustering and stepwise multivariate logistic regression, assessed associations between clinical variables and vascular parameters.

**Results:**

Of 310 young males living with HIV screened, 50 (16.1%) reported erectile dysfunction and were enrolled, with 97 HIV‐negative individuals included as controls. Pathological intima‐media thickness was significantly higher (*p* = 0.004) in young males living with HIV (76%) than in young controls (49%) but comparable to middle‐aged controls (76.1%). Stepwise multivariate logistic regression identified belonging to the young control group, compared to young males living with HIV, as a protective factor against pathological intima‐media thickness (OR 0.353, 95% CI 0.138–0.902, *p* = 0.0295), while increasing age was a significant risk factor (OR 1.09, 95% CI 1.01–1.18, *p* = 0.0247). Relative inhomogeneity of clusters was tested demonstrating that membership in either the people living with HIV or HIV‐negative group was a significant predictor of cluster assignment based on dynamic penile color Doppler echography parameters, independent of age (*p* = 0.0025).

**Discussion and conclusions:**

This study is the first to utilize dynamic penile color Doppler echography to evaluate erectile dysfunction in young males living with HIV, highlighting the association between HIV and early vascular alterations. Clinicians should incorporate routine sexual health evaluations into routinary out‐patients visits, using erectile dysfunction as a potential indicator for further vascular screening and early intervention.

## 1 INTRODUCTION

Antiretroviral therapy (ART) has changed the natural course of HIV infection making the life expectancy of people living with HIV (PLWH) like that of individuals without HIV infection.^1^ However, a large and persistent difference in comorbidity‐free years may still be observed in PLWH living over 16 fewer healthy years than uninfected adults.^1^ According to the UNAIDS “fourth 90,” national and international guidelines are pointing out all the efforts that the HIV expert should take in recognizing and addressing at least the most relevant comorbidities that affect PLWH well‐being.^2^ Sexual health is a primary component of people's health and, therefore, sexual issues contribute to a suboptimal quality of life in PLWH.^3^

Erectile dysfunction (ED) is a multidimensional condition defined as the failure to achieve or maintain a rigid penile erection suitable for a satisfactory sexual intercourse.^4^ The ED prevalence in men living with HIV ranges from 13% to 86%^5^; however, the real entity of the problem might be underestimated. Moreover, several analysis enlighten that these estimates are higher than the age‐matched HIV‐negative population.^6^, ^7^ Immune‐inflammation and adverse ART‐derived reactions play a pivotal role in the pathogenesis of ED in PLWH^8^, ^9^ together with the traditional risk factors that affects also general population (age, lifestyle, cardiovascular, neurological, or endocrine diseases).^5^, ^6^, ^10^, ^11^ Of course, psychological domains dealing with fear of HIV transmission during sexual activity, social stigma, and cultural aspects in general create a vicious circle of sexual dysfunction in PLWH.^5^

However, the specific contribution of metabolic, vascular, hormonal, and psychogenic factors, among PLWH experiencing ED remains insufficiently explored in the current literature. This gap is particularly notable when considering the diagnostic relevance of dynamic penile color doppler echography (dpCDE), as emphasized in the most recent Andrology guidelines.^12^ This methodology is a reliable and well‐established procedure, recognized as the gold standard for diagnosing penile vascular pathologies and their severity.^13^ Moreover, it should be utilized in men requiring precise cardiovascular risk stratification, as PLWH, allowing for the early identification of patients who may benefit from cardiovascular assessment.^13^

This study aims to evaluate the penile vascular status of young (under 50 years old) males living with HIV (yMLWH) presenting with ED through dpCDE. Given that PLWH often experience age‐related comorbidities approximately 10 years earlier than the general population,^14^ the assessment was conducted by comparing this cohort to a control group of HIV‐negative individuals with ED, stratified into two cohorts: young controls (yC, aged under 50 years) and middle‐aged controls (maC, aged 51‒60 years).

## 2 METHODS

### 2.1 Study design and participants

This was a comparative cross‐sectional monocentric pilot study in which we enrolled yMLWH experiencing ED attending our Unit of Infectious Diseases, ASST Spedali Civili di Brescia, Italy, from June 2023 to December 2023. All yMLWH were asked for symptoms of ED during routine follow‐up visits as recommended by the most update HIV management international guidelines.^15^ Inclusion criteria were a documented HIV infection, being on‐ART for more than 12 months, and having an age among 18 and 50 years. We restricted the age range to limit age‐related factors that may contribute to ED. No exclusion criteria were considered. For those who were included, a multidisciplinary evaluation involving a specialist in endocrinology and andrology from ASST Spedali Civili di Brescia was arranged to conduct a dpCDE. A comparative group of HIV‐negative individuals with ED, all under 60 years old, referred to the same Andrology Unit during the same period, was included. This comparative group was stratified by age into two cohorts: yC (under 50 years) and maC (51‒60 years). Written informed consent was collected from every participant.

### 2.2 Sexual function assessment

All yMLWH included were asked for symptoms of ED during the routinary HIV follow‐up visits. In case of referred ED, sexual function was assessed through the International Index of Erectile Function‐5 (IIEF‐5) questionnaire. The most updated European Guidelines for HIV management^15^ recommend the use of IIEF, which is a multidimensional validated questionnaire approved by National Institutes of Health investigating the five domains of sexual function (erectile and orgasmic functions, sexual desire, satisfaction with intercourse, and overall sexual satisfaction).^16^ We used the IIEF‐5 that is a more recent, simplified, easily applicable in clinical practice, and abridged five‐item version that proved to be a valid specific and sensitive scale in the clinical setting.^16^ The possible scores for the IIEF‐5 range from 5 to 25, and we classified ED into five categories based on the following ranges: severe (5–7), moderate (8–11), mild to moderate (12–16), mild (17–21), and no ED (22–25).

### 2.3 Data collection

During the multidisciplinary visit, demographic and anthropometric (weight, height, and body mass index [BMI]) variables were collected. A thorough examination was performed, and vital signs were taken. YMLWH were asked about their past medical history, smoking habits, and the eventual use of phosphodiesterase 5 inhibitors in the past. Comorbidities were defined using the most updated version of EACS guidelines.^15^ A blood count was collected with the following metabolic parameters using commercially available kits: glycemia, creatinine, total cholesterol, high‐density lipoprotein cholesterol, and triglycerides. Low‐density lipoprotein cholesterol was calculated using the Friedwald formula. Blood samples for hormonal analysis were obtained between 08:00 and 10:00 am following a 12‐h overnight fast. The following biochemical assays were performed: total testosterone (TT), prolactin (PRL), sex hormone‐binding globulin (SHBG), follicle‐stimulating hormone (FSH), luteinizing hormone (LH), and estradiol (E2). All assays were conducted using chemiluminescence‐based techniques, specifically chemiluminescence microparticle immunoassay for TT, PRL, FSH, LH, and E2, and chemiluminescence immunoassay for SHBG. Calculated free testosterone (cFT) was calculated based on SHBG and albumin concentration according to the Vermeulen formula.^17^ The intra‐ and inter‐assay coefficients of variation for TT, LH, and SHBG were below 5%. All analyses were performed at the Central Laboratory of the University Hospital of Brescia. The diagnosis of biochemical hypogonadism was determined based on LH and cFT levels following established guidelines.^18^ A cFT value <63 pg/mL was considered low, while normal LH levels were defined as <9.4 IU/L. Hypogonadism was classified in primary (LH > 9.4 IU/L and cFT < 63 pg/mL), normogonadotropic/secondary (LH ≤ 9.4 and cFT < 63 pg/mL), and subclinical/compensated (LH > 9.4 IU/L and cFT ≥ 63 pg/L).^18^, ^19^ TT levels were categorized according to guidelines for functional hypogonadism: TT < 2.3 ng/mL was indicative of hypogonadism, and values between 2.3 and 3.5 ng/mL were considered borderline.^19^, ^20^, ^21^

### 2.4 Penile Doppler ultrasonography

All patients underwent penile dynamic color doppler ultrasound, performed by a single experienced operator using high‐resolution, high‐frequency linear probes (7.5–14 MHz). The procedure involved an intracavernous injection of 10 µg alprostadil, followed by assessment of intracavernous blood flow at the peno‐scrotal junction over a 20‐min period. Tardive response was defined as the achievement of a complete erection occurring after 20 min following the intracavernous administration of alprostadil. Parameters evaluated included peak systolic velocity (PSV) and intima‐media thickness (IMT) of the right and left cavernous arteries to assess for ED secondary to arterial disease.^12^, ^13^ Arterial dysfunction was defined by mean cavernous artery PSV < 35 cm/s or IMT ≥ 0.3 mm in at least one artery.^22^ Veno‐occlusive dysfunction was identified when the mean end‐diastolic velocity (EDV) between the two arteries exceeded 5 cm/s or resistance index (RI) values were <0.9.^12^, ^13^

### 2.5 Ethics

The study protocol was approved by the ASST Spedali Civili Ethics Committee (protocol no. 5184) and conducted in accordance with the ethical standards of the Helsinki Declaration (1975, revised in 2013). Written informed consent has been obtained from each subject.

### 2.6 Statistical analysis

The dataset was stratified into two groups: cases (yMLWH) and controls (HIV‐negative individuals), which were further subdivided by age into yC and maC. Categorical variables were summarized as counts and percentages, while continuous variables were reported as means with standard deviations or medians with ranges (min–max), depending on data distribution continuous nonparametric variables were compared using the Mann–Whitney *U*‐test or the Kruskal–Wallis test, as appropriate for data, whereas categorical variables were analyzed using the chi‐squared test or Fisher's exact test. Using the complete dataset, we performed a *k*‐means cluster analysis, a widely recognized clustering method in the field of machine learning. This analysis employed an unsupervised learning algorithm that was specifically adapted to effectively process categorical data.^23^ This analysis incorporated the dpCDE parameters IMT, PSV, and EDV, categorized as either physiological or pathological (pathological thresholds defined as EDV ≥ 5 cm/s, PSV < 35 cm/s, or IMT ≥ 0.3 mm). Notably, this algorithm permits the predefined specification of the number of clusters. Guided by our clinical hypothesis, we predetermined the number of clusters as two to assess whether the case group and the control group exhibited heterogeneous behavior concerning the parameters under investigation. To evaluate the associations between clinical variables and ultrasound parameters, multivariate logistic regression analyses were performed using the “stepwise” backward and backward/forward methods, including all covariates previously discussed, with IMT, PSV, and EDV considered separately as dependent variables. Results were presented as odds ratios (ORs) with 95% confidence intervals (CIs). Given the limited literature available, a formal sample size calculation was not feasible; therefore, the study was designed as a pilot investigation. All statistical analyses were performed using the R statistical software,^24^ with a significance level set at alpha <0.05.

## 3 RESULTS

During the study period, 310 yMLWH were screened for eligibility, of whom 50 (50/310, 16.1%) reported ED and were subsequently enrolled. All yMLWH included in the study were virologically suppressed (plasmatic HIV‐RNA < 20 cp/mL), with a mean CD4/CD8 ratio of 0.9 ± 0.45. For the control group, 97 HIV‐negative individuals presenting with ED were evaluated at the Andrology Clinic in the same period and included in the study. The control group was stratified into two cohorts: 51 yC and 46 maC. A summary of all demographics, clinical data, and hormonal assays is presented in Table 1.

**TABLE 1:** Demographic, clinical data, and hormonal assays of the included people.

	HIV positive	HIV negative (≤50 years old)	HIV negative (50‒60 years old)	*p*‐value
Demographic				
Sample size, *n* (%)	50 (100)	51 (100)	46 (100)	
Age (years), mean (SD)	44.4 (4.3)	42.3 (7.3)	55.7 (2.8)	**<0.001**
BMI (kg/m^2^), mean (SD)	26.4 (4.4)	25.9 (3.5)^a^	27.8 (4.0)^a^	**0.035**
Questionnaires				
IIEF‐5 score, mean (SD)	14.2 (5.7)	14.8 (4.5)	13.1 (5.7)	0.320
Comorbidities				
Arterial hypertension, *n* (%)	10 (20)^a^	12 (23.5)	21 (45.7)^a^	**0.012**
Dyslipidemia, *n* (%)	9 (18)	2 (4.3)^a^	14 (30.4)^a^	**0.005**
Psychiatric diseases, *n* (%)	8 (16)^a^	2 (4.3)	0 (0.0)^a^	**0.006**
Neurological or pelvical diseases, *n* (%)	4 (8)	6 (13)	6 (13)	0.662
Diabetes, *n* (%)	4 (8)	3 (6.5)	9 (19.6)	0.094
Chronic kidney disease, *n* (%)	2 (4)	1 (2.2)	0 (0.0)	0.396
Currently or previous smoking, *n* (%)	15 (30)	9 (20)	11 (24)	0.491
Hormonal assays				
Hypogonadism, *n* (%)	7 (14)	6 (11.8)	9 (20.5)	0.480
Total testosterone (µg/L), mean (SD)	4.4 (2.1)	5.6 (2.0)	4.7 (1.7)	0.085
SHBG (nmol/L), mean (SD)	38.4 (14.5)	42.1 (22.5)	36.7 (12.4)	0.843
cFT (ng/dL), mean (SD)	94.7 (29.4)	106.2 (28.0)^a^	83.9 (18.6)^a^	**0.007**
FSH (IU/L), mean (SD)	6.7 (5.7)	7.7 (9.7)	6.4 (3.4)	0.636
LH (IU/L), mean (SD)	7.7 (5.2)	6.4 (3.8)	5.7 (2.7)	0.073
PRL (µg/L), mean (SD)	15.8 (15.4)	12.6 (6.5)	28.3 (64.8)	0.310
PSA (µg/L), mean (SD)	1.2 (1.5)	0.8 (0.4)	1.5 (1.4)	0.135

*Note*: Letter (a) denotes statistically significant differences (*p* < 0.05) determined using the Dwass‒Steel‒Critchlow‒Fligner pairwise comparison test.

Abbreviations: BMI, body mass index; cFT, calculated free testosterone; FSH, follicle‐stimulating hormone; IIEF, International Index of Erectile Function; LH, luteinizing hormone; PRL, prolactin hormone; PSA, prostate‐specific antigen; SD, standard deviation; SHBG, sex hormone‐binding globulin.

Results that reach statistical significance are presented in bold type for emphasis.

The maC group exhibited a higher mean BMI (27.8 ± 4.0) and showed a significantly higher prevalence of arterial hypertension (45.7%) and dyslipidemia (30.4%) compared to the yMLWH group (20% and 18%, respectively). Conversely, the prevalence of psychiatric disorders was significantly higher in the yMLWH group (16%) compared to both the yC (4.3%) and maC (0%) groups. Overall, primary or secondary hypogonadism was identified in 14% of yMLWH participants, although this prevalence was not significantly different from that observed in the control groups (11.8% in yC and 20.5% in maC). The mean cFT level was significantly lower in the maC group (83.9 ± 18.6 ng/dL) compared to both the yMLWH group (94.7 ± 29.4 ng/dL) and the yC group (106.2 ± 28.0 ng/dL).

**FIGURE 1:**
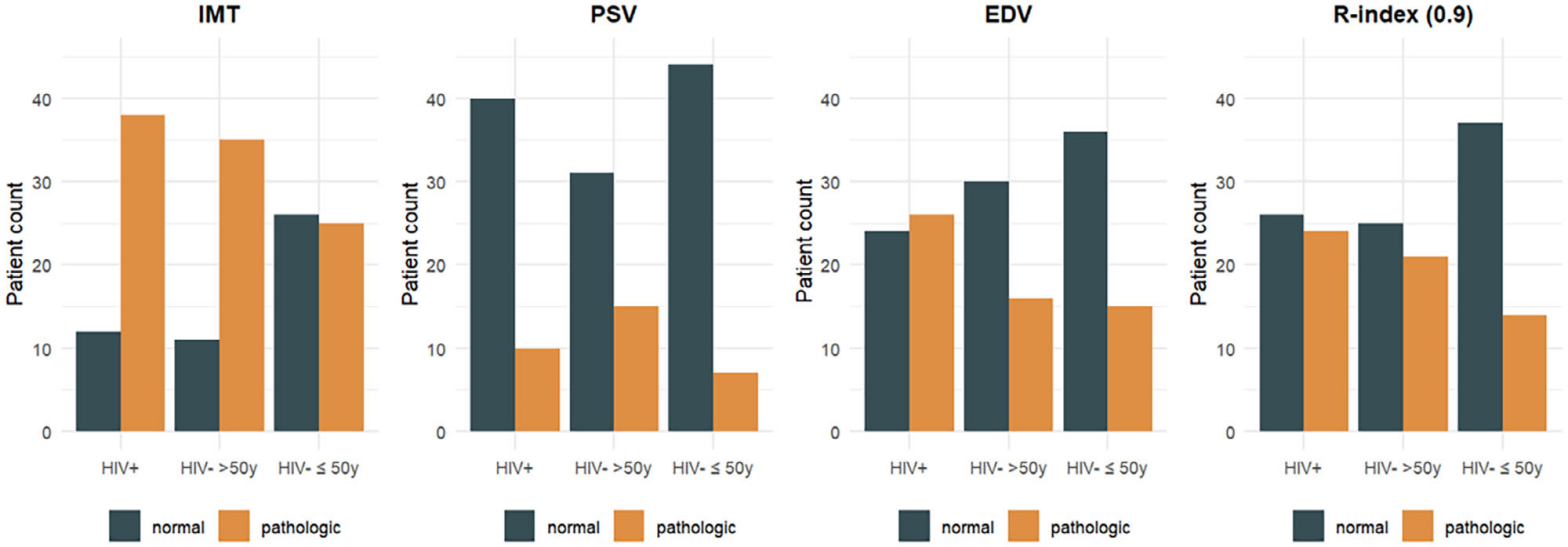
Comparison of intima‐media thickness (IMT), peak systolic velocity (PSV), end‐diastolic velocity (EDV), and resistivity index (RI) across the three groups.

Results of dpCDE assessments are detailed in Table 2. The prevalence of pathological IMT was significantly higher in yMLWH group (76%) and maC group (76.1%) compared to the yC group (49%). Similarly, the prevalence of pathological or tardive response to alprostadil was higher in the yMLWH group (18% and 16%, respectively) compared to HIV‐negative individuals of the same age control group (9.8% and 13.7%, respectively). The mean PSV in the yMLWH group (46.5 ± 16.1 cm/s) was lower compared to the yC group (48.9 ± 14.9 cm/s), but without statistical significance at the pairwise comparison test. The comparison of IMT, PSV, EDV, and RI across the three groups is presented in Figure 1.

**TABLE 2:** Dynamic penile color Doppler echography (dpCDE) assessment of the included people.

	HIV positive	HIV negative (≤50 years old)	HIV negative (50‒60 years old)	*p*‐value
Sample size, *n* (%)	50 (100)	51 (100)	46 (100)	
dpCDE				
Pathological response to injection, *n* (%)	9 (18)	5 (9.8)^a^	15 (32.6)^a^	**0.018**
Tardive response to injection, *n* (%)	8 (16)	7 (13.7)	1 (2.2)	0.068
Pathological IMT, *n* (%)	38 (76)^a^	25 (49)^a,b^	35 (76.1)^b^	**0.004**
Pathological PSV, *n* (%)	10 (20)	7 (13.7)	15 (32.6)	0.074
Pathological EDV, *n* (%)	26 (52)	15 (29.4)	16 (34.8)	0.053
Pathological RI, *n* (%)	24 (16)	14 (9.5)	21 (14)	0.071
IMT, mean (SD)	0.307 (0.076)	0.275 (0.091)^a^	0.339 (0.114)^a^	**0.011**
PSV, mean (SD)	46.5 (16.1)	48.9 (14.9)^a^	41.4 (15.5)^a^	**0.034**
EDV, mean (SD)	4.5 (4.1)^a^	2.6 (3.8)^a^	3.3 (3.5)	**0.048**
RI, mean (SD)	0.882 (0.1)	0.927 (0.1)	0.901 (0.1)	0.087
Cavernous artery tortuosity, *n* (%)	15 (30)	13 (25.5)	20 (43.5)	0.149
Presence of arterial anastomosis, *n* (%)	25 (50)	18 (35.3)	16 (34.8)	0.215
Accessory cavernous arteries, *n* (%)	5 (10)	1 (2)	3 (6.5)	0.240
Penile plaques, *n* (%)	2 (4)	1 (2)	4 (8.7)	0.284

*Note*: Letters (a and b) denote statistically significant differences (*p* < 0.05) determined using the Dwass‒Steel‒Critchlow‒Fligner pairwise comparison test.

Abbreviations: EDV, end‐diastolic velocity; IMT, intima‐media thickness; PSV, peak systolic velocity; RI, resistivity index; SD, standard deviation.

Results that reach statistical significance are presented in bold type for emphasis.

**FIGURE 2:**
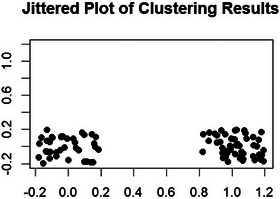
Membership in either the people living with HIV (PLWH) or HIV‐negative group is a significant predictor of cluster assignment based on intima‐media thickness (IMT), peak systolic velocity (PSV), and end‐diastolic velocity (EDV), independent of age.

Relative inhomogeneity of clusters, shown in Figure 2, was tested by multivariate logistic regression, demonstrating that membership in either the PLWH or HIV‐negative group was a significant predictor of cluster assignment based on IMT, PSV, and EDV, independent of age, with statistically significant results (*p* = 0.0025). Notably, the IMT variable was significantly associated with stratification group membership (*p* = 0.004), while PSV and EDV showed only a trend toward significance (*p* = 0.074 and 0.053, respectively).

Stepwise multivariate logistic regression analysis shows that belonging to the yC group compared to the PLWH group was shown to be a protective factor against pathological IMT (OR 0.353, 95% CI 0.138–0.902, *p* = 0.0295). Additionally, almost each additional year of age was identified as a significant risk factor for pathological IMT (OR 1.09, 95% CI 1.01–1.18, *p* = 0.0247) as shown in Table 3.

**TABLE 3:** Backward multivariate stepwise logistic regression analysis for pathological intima‐media thickness.

	OR	95% CI	*p*‐value
Intercept	0.0474	0.00149–1.50	0.0838
Belonging to maC group	0.283	0.0713–1.12	0.0730
Belonging to yC group	0.353	0.138–0.902	0.0295
Age	1.09	1.01–1.18	0.0247
Cardiovascular comorbidities	2.13	0.882–5.16	0.0926
Hypogonadism	2.71	0.724–10.2	0.138

Abbreviations: CI, confidence interval; maC, middle‐aged controls; OR, odds ratio; yC, young controls.

## 4 DISCUSSION

To the best of our knowledge, this study represents the first comprehensive evaluation of ED employing dpCDE in PLWH. Our comparative cross‐sectional analysis suggests that yMLWH experiencing ED exhibit a vascular profile comparable to those observed in HIV‐negative individuals approximately a decade older. Moreover, HIV status resulted as a predictor of clustering based on pathological IMT, PSV, and EDV independently of age. These findings underscore the influence of HIV status on vascular characteristics, emphasizing the importance of considering HIV status in cardiovascular risk assessment. Furthermore, they suggest that HIV itself may serve as an independent factor contributing to early penile vascular alterations.

Among the yMLWH enrolled, 16.1% (50/310) reported ED during the routinary outpatient visits. This observation confirms the wide variability in the reported prevalence of ED among PLWH, which remains highly heterogeneous, ranging from 13% to 86%, even considering the most recent studies.^5^, ^25^, ^26^ The high prevalence within a relatively young population, can be attributed to the frequent association between systemic inflammatory conditions, such as HIV, and ED as outlined in the most updated Italian Andrology guidelines.^12^ Indeed, as illustrated in Figure 2, HIV status emerged as a significant predictor of cluster assignment based on dpCDE parameters.

Although the prevalence of ED in our setting may differ from that observed in other centers, our cohort aligns with the characteristics reported by most European and American centers, where the majority of PLWH are virologically suppressed, exhibit a mean CD4/CD8 ratio close to 1, and are predominantly on integrase inhibitor‐based antiretroviral regimens.^27^

Our yMLWH with ED exhibited a higher prevalence of comorbidities compared to age‐matched controls (yC), with significantly higher rates of psychiatric disorders observed in the yMLWH group compared to both control groups. These findings are consistent with existing literature, which highlights an increased prevalence of major depression, anxiety, bipolar disorder, and schizophrenia among PLWH compared to the general population.^28^, ^29^ Despite this, mental health disorders remain a major global contributor to morbidity and mortality, with limited research on health outcomes in PLWH.^30^ Sexual dysfunction is a common adverse effect of antidepressants, ranging from decreased sexual excitement to ED.^31^ Individuals with severe mental illness face a significantly elevated cardiovascular risk because of biological, behavioral, and systemic healthcare factors, contributing to a markedly reduced life expectancy and disparities in cardiovascular care.^32^ However, despite the high prevalence of psychiatric conditions among yMLWH in our sample, psychiatric disorders did not emerge as a significant independent risk factor for pathological IMT in stepwise multivariate logistic regression analysis. Additionally, primary or secondary hypogonadism was identified in 14% (7/50) of yMLWH, with a higher prevalence observed compared to age‐matched HIV‐uninfected men. While the prevalence of hypogonadism among MLWH has significantly declined in recent years, it remains a common clinical finding in this population.^33^, ^34^

As regards dpCDE, pathological IMT was significantly more prevalent in yMLWH compared to age‐matched controls (yC). This finding underscores the possibility that penile vascular alterations may manifest earlier in yMLWH, paralleling vascular changes observed in other anatomical regions.^35^ This is particularly evident in the context of the carotid artery region; however, in the absence of clinically established cardiovascular disease, PLWH exhibit even impaired coronary microvascular function, independent of the ongoing ART.^36^ As ED is an independent risk factor for major cardiovascular events over the next 3–5 years, it is crucial to identify it in PLWH to implement prevention strategies.^37^ This association has been recently acknowledged in the 2024 Princeton IV consensus guidelines which integrated computed tomography coronary artery calcium scoring into the management of men aged 40–60 years with vascular ED, no prior cardiac history, and an intermediate cardiovascular risk (Atherosclerotic Cardiovascular Disease Risk or ASCVD score 7.5–20%).^38^

Similarly, the mean PSV in yMLWH was lower than that observed in yC, which is particularly concerning given that alterations in PSV typically emerge at later stages in the pathophysiology of ED compared to IMT alterations.^39^ PSV has been thought to be the most accurate indicator of penile arterial disease^40^; however, the absence of statistically significant differences in PSV between yMLWH and yC may be attributed to the fact that this parameter has been demonstrated to be a less reliable diagnostic tool than IMT for the early detection of systemic atherosclerosis,^22^, ^41^ particularly in younger individuals (<50 years old), such as those included in our study cohort. Similarly, the absence of statistically significant differences in EDV may be attributed to the fact that this parameter is less specific and influenced by multiple factors, including reduced arterial elasticity, pelvic floor dysfunction, and suboptimal pharmacological response, rather than being directly associated with age or HIV.^42^ The findings from the logistic regression analysis highlight age as a significant risk factor for pathological IMT. This further supports the inclusion of ED among age‐related comorbidities observed in PLWH, who tend to experience these conditions approximately 10 years earlier than the general population.

Our results should be interpreted in light of several limitations. First, the cross‐sectional design of the study introduces potential biases inherent to this methodology. Second, the epidemiological data are derived from a single‐center cohort, which may limit the generalizability of the findings to broader populations. Last, the pilot nature of the study must be acknowledged, as the lack of established data on dpCDE in case and control groups precluded a formal sample size calculation. Nevertheless, this study also possesses notable strengths. To the best of our knowledge, it is the first to incorporate dpCDE analysis in yMLWH, with both age‐matched and older control groups for comparison. Additionally, the monocentric nature of the study enhances the reliability of the dpCDE data, as all assessments were conducted by the same andrology specialist, ensuring methodological consistency. Laboratory data were similarly standardized, as they were obtained from the same hospital center, minimizing variability. Furthermore, the cohort is representative of real‐life clinical conditions, with all participants being virologically suppressed and maintaining favorable immunological profiles.

## 5 CONCLUSIONS

Our findings highlight that even in a relatively young population of people living with HIV, the presence of erectile dysfunction may indicate underlying vascular alterations. Clinicians, according to the most updated HIV management guidelines, should incorporate routine sexual health evaluations into routinary outpatients visits, using erectile dysfunction as a potential indicator for further vascular screening and early intervention. A multidisciplinary approach, involving andrology specialists, is warranted to facilitate early intervention helping mitigate the risk of more severe cardiovascular events in this at‐risk population. These findings contribute to understanding the early onset of age‐related comorbidities in this population, emphasizing the need for tailored clinical management. Future research with larger sample sizes and longitudinal designs is warranted to establish causal relationships between HIV, vascular alterations, and erectile dysfunction.

## AUTHOR CONTRIBUTIONS

Eugenia Quiros‐Roldan contributed to the study conception and design. Material preparation, data collection, and analysis were performed by Giorgio Tiecco, Andrea Delbarba, Cosimo Colangelo, Marco Di Gregorio, Paolo Facondo, Matteo Riva, and Eugenia Quiros‐Roldan. The first draft of the manuscript was written by Giorgio Tiecco, Andrea Delbarba, Matteo Riva, and Eugenia Quiros‐Roldan. All authors read and approved the final manuscript.

## CONFLICT OF INTEREST STATEMENT

The authors declare no conflicts of interest.

## FUNDING INFORMATION

The authors received no specific funding for this work.
